# Involvement of Muscarinic Receptors in Hypotensive and Diuretic Effects of Aqueous Soluble Fraction from *Asphodelus tenuifolius* Cav.

**DOI:** 10.1155/2021/6653270

**Published:** 2021-01-15

**Authors:** Waqas Younis, Alamgeer, V. B. Schini-Kerth, Samara Requena Nocchi, Denise Brentan Silva, Priscila de Souza, Ishfaq Ali Bukhari, Fahim Vohra, Sibtain Afzal, Arquimedes Gasparotto Junior

**Affiliations:** ^1^Laboratory of Cardiovascular Research and Integrative Pharmacology, College of Pharmacy, University of Sargodha, Sargodha 40100, Pakistan; ^2^Faculty of Pharmacy, The University of Lahore, I-Km Defence Road, Lahore, Pakistan; ^3^Punjab University College of Pharmacy, University of the Punjab, Lahore 54000, Pakistan; ^4^UMR CNRS 7213, Laboratory of Biophotonics and Pharmacology, Faculty of Pharmacy, University of Strasbourg, Illkirch, Strasbourg, France; ^5^Laboratory of Natural Products and Mass Spectrometry, Faculty of Pharmaceutical Sciences, Food and Nutrition, Federal University of Mato Grosso do Sul, Campo Grande 79.070-900, MS, Brazil; ^6^Postgraduate Program in Pharmaceutical Sciences, University of Vale Do Itajaí, Itajaí, SC, Brazil; ^7^Department of Pharmacology, College of Medicine, King Saud University, Riyadh, Saudi Arabia; ^8^Department of Prosthetic Dental Sciences, College of Dentistry, King Saud University, Riyadh, Saudi Arabia; ^9^Immunology Research Center, College of Medicine, King Saud University, Riyadh, Saudi Arabia; ^10^Laboratory of Cardiovascular Pharmacology (LaFaC), Federal University of Grande Dourados (UFGD), P.O. Box 533, Dourados 79.804-970, MS, Brazil

## Abstract

*Background*. *Asphodelus tenuifolius* Cav. (Asphodelaceae) is widely used in Pakistan traditional medicine as a hypotensive and diuretic agent. Despite the cardioprotective effects described for *A. tenuifolius*, the mechanisms involved in its probable hypotensive and diuretic effects have never been evaluated. Firstly, different extracts from *A. tenuifolius* seeds were obtained, and their antioxidant profiles and chemical constituents by LC-DAD-were determined, including molecular networking by the GNPS platform. Then, to evaluate changes in blood pressure, different groups of anesthetized normotensive rats were intravenously treated with the crude extract (AT-Cr, 1–50 mg/kg), aqueous (AS-AT, 1–25 mg/kg), n-butanol (BS-AT, 1–50 mg/kg), and dichloromethane fraction (DS-AT, 1–80 mg/kg). The diuretic effects of AT-Cr, AS-AT, BS-AT, and DS-AT at 100, 200, and 300 mg/kg, p.o. doses, were also evaluated in comparison with hydrochlorothiazide (HCTZ, 10 mg/kg, p.o). The urinary volume, sodium, potassium, and pH were estimated in the sample collected for 6 h from saline-loaded rats. Using pharmacological antagonists or inhibitors, we determine the involvement of acetylcholine, prostaglandins, and nitric oxide in *A. tenuifolius*-induced hypotensive and diuresis action. In addition, the activities of angiotensin-converting enzyme, erythrocyte carbonic anhydrase, and renal Na+/K+/ATPase were evaluated *in vitro*. Acute treatment with crude extract and fractions of *A. tenuifolius* exhibited significant hypotensive and diuretic potential in normotensive rats. However, AS-AT produced the most potent and significant dose-dependent hypotension and diuretic effects in normotensive rats. Previous treatment with atropine significantly reduced the hypotensive and diuretic action of AS-AT, but pretreatment with indomethacin or L-NAME did not affect these effects. Moreover, the 7-day treatment with AS-AT did not reduce activities of serum angiotensin-converting enzyme, erythrocyte carbonic anhydrase, and renal Na+/K+/ATPase. AS-AT showed four major compound node clusters, which included sugars, alkaloids, nucleoside, amino acid, and glycosylated flavonoids. This research supports and extends the traditional use of *A. tenuifolius* as a hypotensive and diuretic agent. The results showed that AS-AT from *A. tenuifolius* could present compounds responsible for hypotensive and diuretic activities through the activation of muscarinic receptors.

## 1. Introduction

Cardiovascular disorders (CVDs) are continuously increasing in patients with renal insufficiency leading to their high disease and death rates [1]. Several problems in kidney functions become prerequisites for hypertension. 70–80% of chronic kidney disease (CKD) patients also have hypertension because problems in tubular reabsorption of sodium and water in chronic kidney diseases may lead to fluid accumulation and hypertension [2, 3]. High salt intake increases the vascular tone and suppresses nitric oxide release. Besides that, high sodium levels also augment the potential effects of angiotensin II and norepinephrine [4].

In cardiovascular complication, diuretic agents as loop diuretics, thiazide, and potassium-sparing diuretics are commonly considered to mitigate the exacerbation related to congestion and edema. Diuretics today are a well-reputed medication for the reduction of blood volume and venous pressure to treat essential hypertension and heart failure. In addition to their beneficial role in hypertension, diuretics also improve mortality associated with chronic kidney disease, pulmonary edema, stroke, and congestive heart failure [5]. Meanwhile, pharmacovigilance clinical data also assure that diuretics are commonly related to a variety of adverse effects, such as alteration in systemic electrolyte balance, hypovolemia, metabolic alkalosis or acidosis, and hyperuricemia [6]. So, in the era of modern medicine, it is necessary to design and develop new diuretic agents that not only improve the therapeutic outcome in cardiovascular morbidities but may also overcome the undesired effects and events.

Recently, the bulk of studies reported on the pharmacological potential to explore and introduce new diuretic agents from natural products. These reports are mostly based on the empirical utilization of these herbs [7]. In Pakistan, a variety of herbal medicines are considered and traditionally being used as a diuretic, but an important need is to establish their folkloric claim on behalf of modern medicines.

*Asphodelus tenuifolius* Cav. (Asphodelaceae) is an annual, wild, herbaceous species locally named as bhokal, piazi, asphodel, and onion-like weed [8]. It is mostly observed in the Mediterranean area, North Africa, southern Europe, India, and Pakistan [9]. It is an edible plant generally considered as a cultivated vegetable [10]. *A. tenuifolius* seeds are majorly considered for the treatment of different cardiovascular diseases such as hypertension and diabetes. Various ethnobotanical studies have reported that seeds are diuretic in nature and can be used as a part of condiment or spice for the treatment of hypertension [8, 11]. Seeds are also recommended for irritable bowel and digestive complications including hemorrhoids. It is also considered for the treatment of rheumatic pain and other inflammatory joint problems [12–14]. Phytochemical analysis of different extracts after cold and hot extraction techniques has confirmed the presence of polyphenols, chromones, condensed tannins, anthocyanin, anthraquinones glycosides, and a variety of alkaloids. Moreover, various phytochemical studies have evidenced the presence of chlorogenic acid, caffeic acid, vanillin, apigenin, chrysoberyl, rutin, and luteolin [9, 15].

A previous research study from our laboratory has revealed that *A. tenuifolius* has beneficial effects in reducing blood pressure and vascular complication, as well as protective effects on insulin resistance, dyslipidemia, and oxidative stress in glucose-fed rats [15]. Moreover, *A. tenuifolius* has also been studied for antimicrobial, anti-inflammatory, antioxidant, diuretic, and lipoxygenase inhibitory activity [9, 10]. Therefore, considering the traditional uses and previous evidence of the effectiveness of *A. tenuifolius* against cardiovascular diseases, the current research was carried out to investigate the molecular mechanisms involved in the possible diuretic effect of *A. tenuifolius* and verify its relationship with a potential hypotensive effect using normotensive rats.

## 2. Materials and Methods

### 2.1. Chemicals and Drugs

Methanol, n-hexane, dichloromethane, ethyl acetate, n-butanol, hydrochlorothiazide (HCTZ), acetazolamide (ACTZ), N-*ω*-Nitro-L-arginine methyl ester (L-NAME), indomethacin, atropine, ouabain octahydrate, hexamethonium bromide, captopril, and propranolol were purchased locally from Sigma-Aldrich Chemicals. Acetonitrile and formic acid were obtained from J. T. Baker. All these chemicals were of standard analytical grade.

### 2.2. Animals

Sprague-Dawley rats (220–250 g) from the central vivarium of the University of Sargodha were used in this study. All animals were maintained in controlled conditions (12 h light/dark cycle, 25 ± 1°C temperature) with feed and water ad libitum. Animals were provided with housing conditions following accepted principles for laboratory animal use and care (NIH publication number # 85–23, revised in 1985), and we try to minimize animal distress and the number of animals used. All experimental procedures were approved (No. IAEC/UOS/2016/46) by the Ethical Committee of College of Pharmacy, University of Sargodha.

### 2.3. Extraction and Fractionation of Plant Material

The seeds of *A. tenuifolius* were purchased from a local herbal market in Lahore, Pakistan. Seeds were identified and authenticated by a plant taxonomist Dr. Amin-Ullah Shah, and a voucher specimen (#W-6031) was submitted for further research reference at the Herbarium of the University of Sargodha. *A. tenuifolius* seeds (4 kg) were shade dried and pulverized to a coarse constituency for extraction. The powder was soaked in 3 L water and methanol (30 : 70, v/v), followed by filtration with a muslin cloth and filter papers. This process was repeated three times, and the filtrate was concentrated by using a rotary evaporator and stored in a cool place (4–6°C). The percentage yield of this crude extract of *A. tenuifolius* (AT-Cr) was 11%. For activity guided fractionation, 100 g AT-Cr was mixed with distilled water, and liquid-liquid extractions were performed using solvents with different polarity based absolute solvents: hexane, dichloromethane, ethyl acetate, and n-butanol. Obtained fractions were concentrated at 40°C using a rotary evaporator. This process resulted in 26.4 g of the hexane fraction (HS-AT), 7 g of the dichloromethane fraction (DS-AT), 22.4 g of the butanol fraction (BS-AT), and 43 g of the aqueous soluble fraction (AS-AT). The ethyl acetate fraction was obtained in negligible quantities. The hexane soluble fraction was not used because it is insoluble in employed solvents. All these samples were placed at 4°C in a refrigerator for further pharmacological and phytochemical studies [16].

### 2.4. Identification of Constituents by LC-DAD-MS Analysis and Molecular Networking

Previously, the samples DS-AT, BS-AT, and AS-AT were analyzed by LC-DAD-MS, and here, they were reanalyzed to perform annotation by GNPS and to create the molecular network. A UFLC Prominence Shimadzu coupled to diode array detector (DAD) and a mass spectrometer MicrOTOF-Q III (Bruker Daltonics, Billerica, MA, USA) was used. The analyses were performed by a Kinetex C18 column (2.6 *μ*m, 150 × 2.1 mm, Phenomenex) applying the flow rate of 0.3 mL/min and oven temperature of 50°C. Ultrapure water (solvent A) and acetonitrile (solvent B), both added to 0.1% formic acid (v/v), were used as mobile phase. The applied methods were the same described by Tolouei et al. (2019) [17]. The samples were prepared at 4 mg/mL, and 2 *μ*L was injected into the chromatography column.

The annotation of the chemical constituents was performed by comparison of spectral data (UV, MS, and MS/MS) with published data, as well as by the GNPS platform (https://gnps.ucsd.edu/). To create the molecular network, six fragment ions were considered, and for precursor and fragment ions, the mass tolerances were considered up 0.02 Da and 0.1 Da. The molecular network was based on edges with cosine scores of more than 0.7 and 6 ions [18]. The molecular network was visualized in software Cytoscape 3.8.

### 2.5. Determination of Total Flavonoid/Phenolic Contents and Antioxidant Potential

For the determination of the total flavonoid contents (TFCs), total phenolic contents (TPC), and antioxidant potential, we utilized the methodology described by Younis et al. (2017) [15]. DPPH (1, 1-diphenyl-2-picryl-hydrazyl) radical scavenging assay, nitric oxide scavenging assay, total reducing power (TRP), and total antioxidant capacity (TAC) assays were used to determine antioxidant characteristics of AT-Cr, AS-AT, BS-AT, and DS-AT.

### 2.6. Direct Measurement of Blood Pressure

The direct surgical method was employed for measuring BP in anesthetized normotensive rats using thiopental (70–90 mg/kg). The trachea was exposed and cannulated to improve respiration during the length of the experiment. Next jugular vein and carotid artery were exposed and cannulated using a polyethylene catheter (PE-50) for extract/drug administration and recording BP, respectively. A catheter inserted into the carotid artery was connected to the pressure transducer fixed with the PowerLab data acquisition system for recording BP (ADI Instruments; Castle Hill, Australia). Following surgery, animals were allowed to stabilize for 30 minutes prior to record BP [19].

#### 2.6.1. Hypotensive Dose-Response Relationship of *A. tenuifolius* Extracts

Rats were separated into five groups (*n* = 6): Group I = received NaCl 0.9%, Group II = AT-Cr (1–50 mg/kg), Group III = BS-AT (1–50 mg/kg), Group IV = AS-AT (1–25 mg/kg), and Group V = DS-AT (1–80 mg/kg). Blood pressure and heart rate were estimated for 45 minutes after dosing. Freshly prepared dilutions of extract were administered to rats at 1 ml/kg, and a dose of the most active fraction was selected which provided a 50% reduction in BP to further understand the blood pressure-lowering mechanism.

#### 2.6.2. Evaluation of Mechanisms Underlying the Hypotensive Effect of *A. tenuifolius*

Normotensive rats were anesthetized using thiopental (70–90 mg/kg), and blood pressure was measured using the direct surgical method as described earlier. Different groups of rats were given hexamethonium bromide (30 mg/kg), atropine (1 mg/kg), captopril (2.5 mg/kg), indomethacin (5 mg/kg), L-NAME (20 mg/kg), and propranolol (100 mg/kg), 10 min prior to AS-AT (25 mg/kg, i.v) administration. Change in blood pressure was documented for 45 min postdosing [19].

### 2.7. Assessment of the Diuretic Effect

The method used by Kau et al. [20] was followed by a few modifications to access the diuretic activity of extract/fractions. Rats were divided into eleven groups (*n* = 5) for acute study and three groups (*n* = 5) for a 7-day prolonged study. Animals fastened for 12 hours before experiment with access to water *ad libitum*. Animals were acclimatized for one week by placing them in separate individual metabolic cages daily for environmental adaptation.

#### 2.7.1. Acute Diuretic Activity

To assure uniform water and sodium load, saline (0.9% NaCl) at 5 ml/100 gm was given to the animals 45 min before extract administration. The control group was given vehicle (deionized water) and standard group 10 mg/kg HCTZ while treatment groups were given 100, 200, and 300 mg/kg of AT-Cr, BS-AT, AS-AT, and DS-AT. Instantly after dosing, animals were kept in metabolic cages, and urine was collected and volume was recorded at 2, 4, and 6 h. Cumulative urine excretion was calculated according to body weight as mL/100 g. At the end of the experiment, urine electrolyte concentration (sodium and potassium) was estimated and expressed as mmol/L; moreover, urine pH was also determined [21].

#### 2.7.2. Prolonged Diuretic Activity

Overnight fasting animals were given AS-AT (300 mg/kg) for continuous 7 days. A urine sample was collected on the first and seventh days in a graduated cylinder for 6 hours. Urine volume, sodium, and potassium concentration were estimated. Blood was collected by cardiac puncture, and serum was obtained by centrifugation (2000 rpm, 10 mins, 4°C) to measure the plasma concentration of sodium and potassium by an automated electrolyte analyzer [22].

#### 2.7.3. Evaluation of Mechanism Involved in the Diuretic Activity of *A. tenuifolius*

(1) *Role of the Nitric Oxide (NO), Prostaglandins, and Acetylcholine in the Diuretic Effect of A. tenuifolius*. The procedure described earlier for acute activity was followed. Different groups of rats were given L-NAME (60 mg/kg), indomethacin (5 mg/kg), and atropine (1 mg/kg) p.o. 1 h prior to experiments, followed by administration of deionized water (5 ml/kg; p.o.) in the control group and 300 mg/kg AS-AT in the treated group. The urine was collected for 6 h after the treatments. Total urine output as well as urine sodium and potassium concentration was measured [22].

(2) *Measurement of Angiotensin-Converting Enzyme (ACE) Activity in the Prolonged Diuretic Activity*. For ACE activity, rats were separated into four groups (*n* = 5) and treated with AS-AT (30, 100, and 300 mg/kg) and captopril (60 mg/kg). Briefly, after seven days of treatment, blood was collected from overnight fasting treated animals and centrifuged, and serum was separated. Serum (10 *μ*L) was mixed with assay solution (490 *μ*L) and incubated for 15 min at 37°C. Then 1.2 mL of NaOH was added to halt the reaction followed by the addition of 100 *μ*L of o-phthaldialdehyde. Finally, the formation of His-Leu in the reaction mixture was analyzed fluorometrically in triplicate to measure ACE activity.

(3) *Measurement of Erythrocyte Carbonic Anhydrase Activity in the Prolonged Diuretic Activity*. Rats were separated into four groups (*n* = 5) and treated with AS-AT (30, 100, and 300 mg/kg) and acetazolamide (10 mg/kg). Briefly, after 7 days, blood samples were collected, and red blood cells (RBCs) were isolated. 1 mL of RBCs was added to 3 mL of distilled water and 3 ml of chloroform. This mixture was centrifuged for 10 min at 8000 g to obtain supernatant which was diluted in distilled water at the ratio of 1 : 10 (v/v), forming a hemolysate at a dilution of 1/40 (v/v). Two samples of hemolysate (50 *μ*L) were incubated with 1 ml of alpha-naphthyl acetate containing 2% dioxane. One sample was incubated at 37°C with 1 ml acetazolamide for 20 min whereas, in the 2nd sample, acetazolamide was added at the end of incubation followed by the addition of 500 *μ*L of 5-chloro-o-toluidine to both samples. The reaction mixture was kept for 15 minutes followed by measurement of absorbance at 555 nm in triplicate [23].

(4) *In Vitro Determination of Renal Na*^*+*^*/K*^*+*^*/ATPase Activity*. The Na+/K+/ATPase activity was estimated by the methodology of Noel and Godfraind [24], with minor changes using kidney samples with increasing concentrations (3–30 *μ*M) of AS-AT. The assay solution (0.5 ml) was incubated into the kidney samples for 2 hr at 37°C. Before incubation, proteins in the kidney were so adjusted so that only 10–15% of the substrate could be hydrolyzed. The specific activity of the Na+/K+/ATPase enzyme was measured by the difference in the ATPase activity in the absence and presence of 1 mM ouabain (ouabain-resistant activity).

### 2.8. Statistical Analysis

The results were statistically analyzed by GraphPad prism 5.0 and presented as mean ± standard error mean (SEM). One-way ANOVA and two-way ANOVA were applied as required followed by Dunnett's or Bonferroni's posttest whereas *P* values less than 0.05 were considered as significant.

## 3. Results

### 3.1. Annotation and Molecular Networking of the Constituents from *A. tenuifolius*

**Figure 1:**
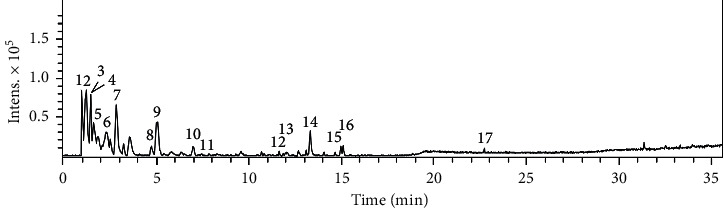
Base peak chromatogram (positive ion mode) from the aqueous soluble fraction of *A. tenuifolius*.

**Figure 2:**
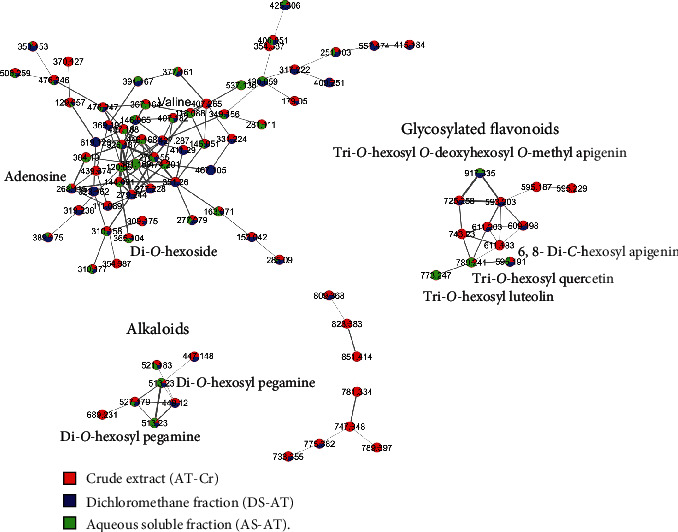
Molecular network from *A. tenuifolius* samples (AT-Cr, DS-AT, and AS-AT).

The extract and fractions from *A. tenuifolius* were previously analyzed by LC-DAD-MS, and some constituents could be annotated [25]. Thus, we reanalyzed them to expand the annotation of the constituents using GNPS library and data reported in the literature (Figure 1, Table 1); moreover, the data were applied to create the molecular network from their constituents and so determine the goal compounds from the most active fraction AS-AT (Figure 2). The molecular formulae of the constituents from AS-AT were determined based on the mass errors up ±10 ppm and mSigma below 30.

The peaks **1, 2,** and **3** were previously described from *A. tenuifolius* [25], and they were putatively annotated as hexitol (*m/z* 183.0861 [M+H]^+^, C_6_H_14_O_6_), di-O-hexoside (*m/z* 365.1037 [M+Na]^+^, C_12_H_22_O_11_), and tri-O-hexoside (*m/z* 527.1554 [M+Na]^+^, C_18_H_32_O_16_). The compounds **4** and **6** revealed protonated ions at *m/z* 118.0863 and 268.1056 relative to molecular formula C_5_H_11_NO_2_ and C_10_H_13_N_5_O_4_. Their fragment ions *m/z* 101 and 136 were yielded from losses of NH_3_ (17 *u*) and pentosyl group (132 *u*). Thus, they were annotated as the amino acid valine (**4**) and the nucleoside adenosine (**6**) [26].

The peaks **8** and **9-10** revealed similar UV spectra (*λ*_max_ ≈ 285 nm). The fragment ions at *m/z* 171 [M + H-H_2_O]^+^ from **8** and *m/z* 351 [M + H-hexosyl]^+^ and 189 [M + H-2 × hexosyl]^+^ from **9**/**10** were compatible with the observed for peganine and di-*O*-hexosyl pegamine, respectively [27, 28]. In addition, the compounds **12-13**, **15**, and **17** exhibited similar UV spectra of flavonols (*λ*_max_ = 265 and 355 nm) and flavones (*λ*_max_ ≈ 270 and 340 nm) [29]. The precursor ions of **12**, **13**, and **17** revealed fragment ions yielded from subsequent losses of hexosyl groups (162 *u*), such as the ions *m/z* 449 [M + H-hexosyl]^+^ and 287 [M + H-2 × hexosyl]^+^ from **13**. For flavonoid **17**, an additional loss of 146 *u* (*m/z* 431 ⟶ 285) suggested the deoxyhexosyl substituent, and a loss of radical methyl (^•^CH_3_, 15 *u*) from aglycone ion (*m/z* 285 ⟶ 270) suggested the aglycone *O*-methyl apigenin. The aglycone ions of **12** and **13** were observed at *m/z* 303 and 287, respectively. Thus, **12**, **13**, and **17** were annotated as tri-*O*-hexosyl quercetin, tri-*O*-hexosyl luteolin, and tri-*O*-hexosyl *O*-deoxyhexosyl *O*-methyl apigenin [30].

The flavone **15** revealed a similar fragmentation pathway of *C*-glycosylated flavonoid. So, fragment ions yielded from consecutive losses of 90 and 120 *u* suggested the *C*-hexosyl substituents, such as the ions at *m/z* 505 [M + H-90]^+^, 475 [M + H-120]^+^, and 355 [M + H-120-120]^+^. This compound was annotated as 6,8-di-*C*-hexosyl apigenin [31].

The molecular network of the constituents from *A. tenuifolius* exhibited four major node spectral families (Figure 2). The connections (edges) of nodes were represented by lines with thickness relative to cosine score, and the color green in nodes is relative to ion intensities from the AS-AT sample. We can observe a node cluster family of glycosylated flavonoids, but only four flavonoids were observed in AS-AT. In addition, a node cluster was observed from alkaloids.

### 3.2. Total Phenolic (TPC), Flavonoid Contents (TFCs), and Antioxidant Potential of *A. tenuifolius*

Standard regression lines for gallic acid were used to measure total phenolic contents, whereas standard regression lines for quercetin were used to measure total flavonoid contents (Table 2). Phenolic contents in AT-Cr were found as 256.64 ± 4.82 *μ*g gallic acid equivalent/mg, whereas flavonoid contents in ATC-Cr were found as 137.20 ± 2.37 *μ*g quercetin equivalent/mg. The maximum quantity of both polyphenols and flavonoids was found in a crude extract of *A. tenuifolius* as compared to fractions whereas the total reducing power was found almost equal to total antioxidant capacity in all the extract/fractions. Furthermore, AT-Cr also exhibited high TRP and TAC in comparison to other fractions. The % scavenging ability of DPPH and nitric oxide (NO−) by extract/fractions of *A. tenuifolius* is presented in Table 2. The dose-dependent scavenging ability of DPPH and nitric oxide free radicals was observed with all the extract/fractions. Crude extract at the dose of 500 *μ*g/mL showed a maximum of 75.55% inhibition of DPPH radical, whereas a maximum of 66.19% nitric oxide (NO−) free radical inhibition by AT-Cr was observed at IC50 259.4 ± 4.93 *μ*g/mL as compared to ascorbic acid IC50 of 225.81 ± 5.24 *μ*g/mL. Similar DPPH and NO scavenging activities were exhibited by fractions of *A. tenuifolius*. These results indicated that crude extract and fractions of *A. tenuifolius* have significant free radicals scavenging ability.

### 3.3. Hypotensive Effect of *A. tenuifolius* and Its Mechanism of Action in Anesthetized Rats

#### 3.3.1. Acute Hypotensive Effect of *A. tenuifolius* Extracts/Fractions in Normotensive Rats

**Figure 3:**
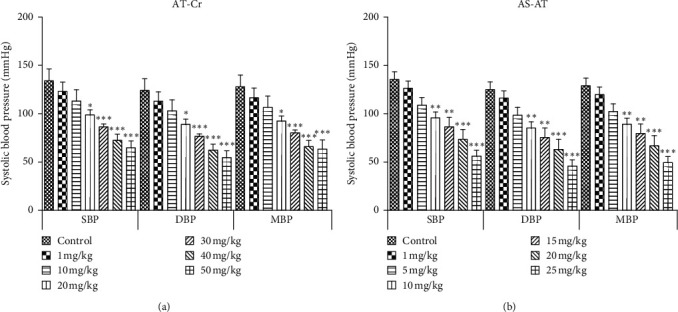
Effect of various doses of (a) *Asphodelus tenuifolius* crude extract (AT-Cr) and (b) aqueous soluble fraction of *A. tenuifolius* (AS-AT) in anesthetized normotensive rats on systolic blood pressure (SBP), diastolic blood pressure (DBP), and mean arterial pressure (MAP). Results are stated as mean ± SEM, whereas ^*∗*^ = *P* < 0.05 and ^*∗∗∗*^ = *P* < 0.001 when compared to the normal saline-treated group.

**Figure 4:**
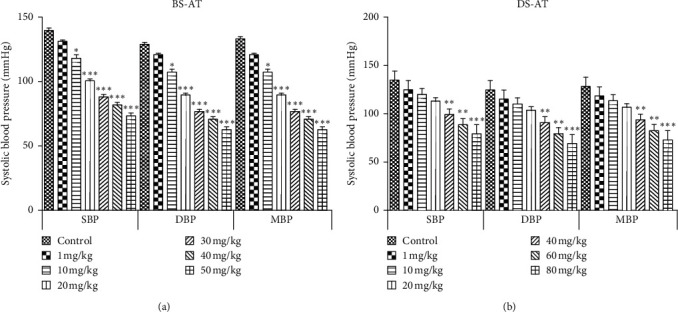
Effect of various doses of (a) butanol soluble fraction of *A. tenuifolius* (BS-AT) and (b) dichloromethane soluble fraction of *A. tenuifolius* (DS-AT) in anesthetized normotensive rats on systolic blood pressure (SBP), diastolic blood pressure (DBP), and mean blood pressure (MBP). Results are stated as mean ± SEM, whereas ^*∗*^ = *P* < 0.05 and ^*∗∗∗*^ = *P* < 0.001 as compared to the normal saline-treated group.

After 30 minutes of stabilization, the average SBP of rats was recorded as 134.68 ± 2.23 mm, and no significant change was observed in blood pressure after the administration of normal saline (vehicle). The administration of crude extract and fractions of *A. tenuifolius* resulted in a significant and rapid reduction of SBP, DBP, and MBP (Figures 3 and 4), but no significant difference in heart rate was observed before and after extract/fractions administration. The intravenous administration of AT-Cr (1, 10, 20, 30, 40, and 50 mg/kg) produced a dose-dependent decline in SBP which lasted for 48.12 ± 3.65, 58.14 ± 2.94, and 70.01 ± 2.14 mm Hg (30, 40, and 50 mg/kg, respectively). Similarly, graded doses of BS-AT (1, 10, 20, 30, 40, and 50 mg/kg) and DS-AT (1, 10, 20, 40, 60, and 80 mg/kg) also produced a dose-dependent decline in SBP (Figure 3.33), which lasted for 66.21 ± 4.19 mm Hg decreases in SBP at 50 mg/kg of BS-AT, and 55.39 ± 1.74 mm Hg decreases in SBP at 80 mg/kg of for DS-AT. In contrast, intravenous administration of AS-AT (1, 5, 10, 15, 20, and 25 mg/kg) caused a highly significant decline in SBP, DBP, and MBP, which lasted for the maximum decrease in SBP for 79.61 ± 2.58 mm Hg at the dose of 25 mg/kg. Based on the most potent hypotensive action with AS-AT at 25 mg/kg dose, it was selected and further used for investigating the mechanism underlying its hypotensive effect.

#### 3.3.2. AS-AT Produced Hypotensive Effect Mediated through Muscarinic Receptors

**Figure 5:**
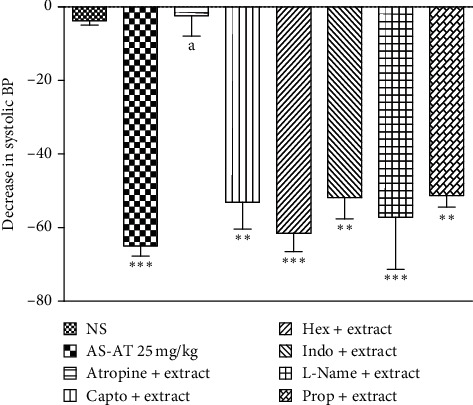
Effect of the aqueous soluble fraction of *A. tenuifolius* (AS-AT, 25 mg/kg) on systolic BP in rats pretreated with various antagonists. Results are stated as mean ± SEM, whereas ^*∗∗*^ = *P* < 0.01 and ^*∗∗∗*^ = *P* < 0.001 as compared to normal saline- (NS-) treated group, while *a* = *P* < 0.001 when compared to the extract-treated control. The figure shows a decrease in systolic BP with AS-AT (25 mg/kg) in anesthetized rats pretreated with normal saline (NS 1 ml/kg), hexamethonium (30 mg/kg), atropine (2 mg/kg), captopril (2.5 mg/kg), indomethacin (5 mg/kg), L-Name (20 mg/kg), and propranolol (Prop 1 mg/kg).

Pretreatment with atropine significantly prevented AS-AT-induced hypotensive effect in normotensive rats. However, propranolol, hexamethonium, L-NAME, indomethacin, and captopril did not change the hypotensive effect of AS-AT. Treatment with only AS-AT (25 mg/kg) causes a 65.27 mmHg decrease in SBP whereas pretreatment with atropine significantly reduced this decrease in SBP to only 2.4 mm Hg (Figure 5). These findings suggested that the hypotensive effect of AS-AT could be mediated by muscarinic receptors.

### 3.4. Results of Diuretic Studies of *A. tenuifolius* Extract/Fractions

#### 3.4.1. Acute Diuretic Effect

(1) *Effect of A. tenuifolius Extract/Fractions on Urine Volume*. Acute diuretic effect with AT-Cr (100, 200, and 300 mg/kg), AS-AT (100, 200, and 300 mg/kg), BS-AT (100, 200, and 300 mg/kg), DS-AT (100, 200, and 300 mg/kg), and hydrochlorothiazide (10 mg/kg) is presented in Tables 3 and 4. AT-Cr (300 mg/kg), BS-AT (300 mg/kg), and DS-AT (300 mg/kg) increased the urine output at 6th h after the treatment whereas AS-AT (200 and 300 mg/kg) produced a highly significant increase in urinary output after 4 and 6 h of treatment. Also, the total urine volume calculated at 4 and 6 h in 300 mg/kg AS-AT treated rats was 4.96 ± 0.75 and 7.19 ± 0.7 mL/100 g, respectively, whereas the urinary output of rats in the control group after 4 and 6 hours was 2.39 ± 0.05 and 3.51 ± 0.12 mL/100 g, respectively. Moreover, urinary output at 4 and 6 hours in rats treated with AS-AT (300 mg/kg) was very similar to the output in rats treated with HCTZ, a standard diuretic drug. Urinary output data of various fractions also showed that urine excretion was increased as the polarity of the solvent increased suggesting that aqueous soluble fractions contain various polar compounds responsible for this activity.

(2) *Effect of A. tenuifolius Extract/Fractions on Electrolyte Excretion*. Effects of treatment with HCTZ (10 mg/kg), AT-Cr (100–300 mg/kg), AS-AT (100–300 mg/kg), BS-AT (100–300 mg/kg), and DS-AT (100–300 mg/kg) on the excretion of sodium and potassium are presented in Tables 3 and 4. AT-Cr, at 200 and 300 mg/kg, caused a significant increase in the urinary sodium and potassium excretion whereas BS-AT and DS-AT only enhance the urinary sodium excretion at its maximum dose (300 mg/kg), with a very slight effect on potassium excretion. In contrast, AS-AT at 200 mg/kg caused a significant increase in sodium excretion with a very low effect on potassium excretion, while, at 300 mg/kg, it caused a highly significant (*P* < 0.001) increase in both sodium and potassium urinary excretion. In fact, sodium and potassium excretion induced by AS-AT was 221 ± 18 and 66 ± 11, while in the control group, it was 113.6 ± 13 and 40 ± 1.2 mL/100 g/6 h. Moreover, AS-AT-induced electrolyte excretion was not different than HCTZ-induced electrolyte excretion. These findings present notable parallelism in sodium and urine excretion. Finally, the pH values in all the groups were not different than the control group. Based upon the highly significant increase in urine output and electrolyte excretion, AS-AT (300 mg/kg) was selected for prolonged treatment and for evaluating the mechanism underlying its diuretic activity.

### 3.5. Prolong Diuretic Activity of AS-AT

**Figure 6:**
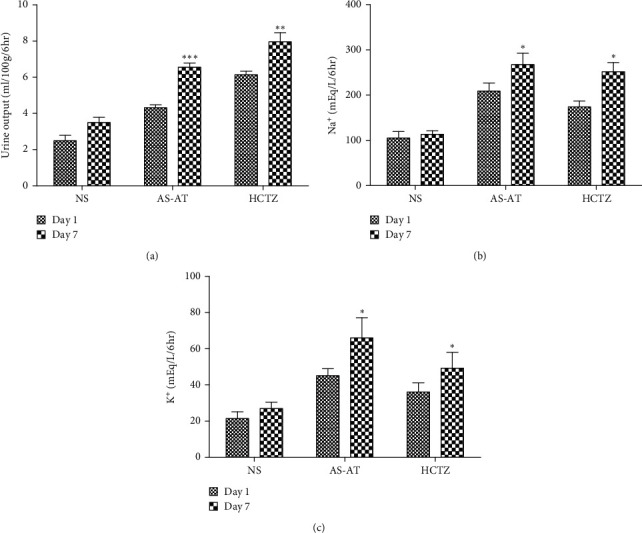
Effect of daily administration of aqueous soluble fraction of *A. tenuifolius* (AS-AT 300 mg/kg) for 7 days on (a) urine output, (b) Na^+^ excretion, and (c) K^+^ excretion. Results are stated as mean ± SEM, whereas ^*∗∗*^ = *P* < 0.01 and ^*∗∗∗*^ = *P* < 0.001 when compared to the normal saline- (NS-) treated group. All data are subjected to two-way ANOVA followed by Bonferroni posttest. HCTZ = hydrochlorothiazide.

Treatment with AS-AT (300 mg/kg) for continuous 7 days significantly increased the urine output (Figure 6). There was also a significant increase in sodium and potassium excretion on the 7th day after treatment with 300 mg/kg AS-AT. These results were very similar to classical diuretic HCTZ.

### 3.6. Mechanism Underlying the Diuretic Potential of AS-AT

#### 3.6.1. AS-AT-Induced Diuresis through Muscarinic Receptors

**Figure 7:**
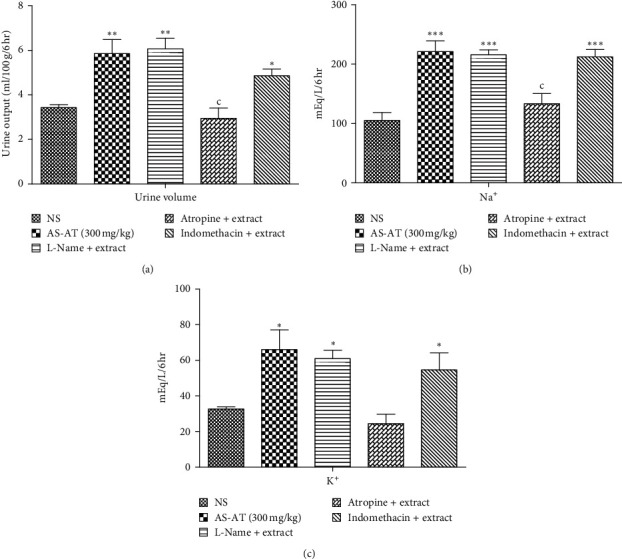
Effect of the aqueous soluble fraction of *A. tenuifolius* (AS-AT 300 mg/kg) on (a) urine output (b) Na^+^ excretion, and (c) K^+^ excretion in the presence of various antagonists. Results are stated as mean ± SEM, whereas ^*∗*^ = *P* < 0.05 and ^*∗∗∗*^ = *P* < 0.001 when compared to the normal saline-treated group, and *c* = *P* < 0.05 when compared to the treated group (AS-AT). Rats in the control group received normal saline (5 mL/100 g), while in all other groups, the animals received normal saline (NS 5 mL/100 g), L-Name (60 mg/kg), atropine (1 mg/kg), and indomethacin (10 mg/kg) 1 hour prior to administration of AS-AT (300 mg/kg).

Pretreatment with atropine significantly prevented the AS-AT-induced diuresis and electrolyte excretion (Figure 7) whereas L-NAME and indomethacin did not alter the ability of AS-AT to induce diuresis.

#### 3.6.2. AS-AT Did Not Inhibit Angiotensin-Converting Enzyme (ACE), Erythrocyte Carbonic Anhydrase, and Renal Na+/K+/ATPase Activity

**Figure 8:**
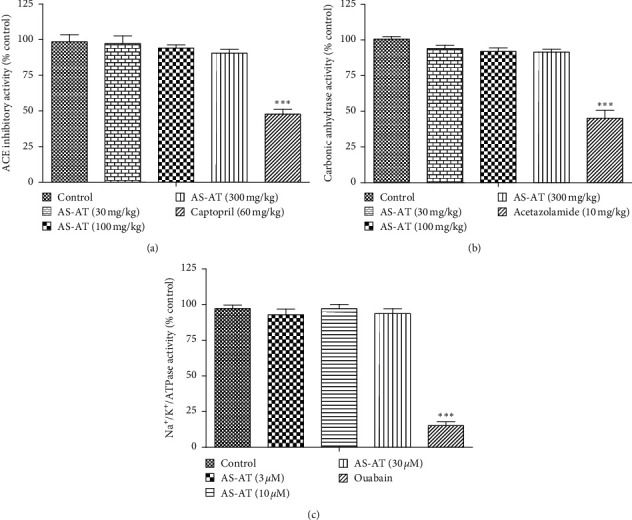
Effect of the aqueous soluble fraction of *A. tenuifolius* (AS-AT) on (a) ACE inhibitory activity, (b) erythrocyte carbonic anhydrase activity, and (c) renal Na^+^/K^+^/ATPase activity. Results are stated as mean ± SEM, whereas ^*∗∗∗*^ = *P* < 0.001 when compared to normal saline-treated group (control).

Treatment with AS-AT did not inhibit ACE activity whereas standard ACE inhibitor, captopril, inhibited the ACE activity around 53% (Figure 8(a)). Treatment with AS-AT did not alter the erythrocyte carbonic anhydrase activity whereas typical carbonic anhydrase inhibitor, acetazolamide, inhibited it by 55 ± 5% (Figure 8(b)). Moreover, AS-AT (3–30 *μ*M) did not change *in vitro* renal Na+/K+/ATPase activity (Figure 8(c)).

## 4. Discussion

Hypertension has been a global health problem owing to its recurrent incidence, incessant, and uncontrolled threat for associated cardiovascular and kidney diseases [32]. According to an estimation by the world health organization (WHO), presently, 17.1 million deaths are happening each year worldwide due to different CVDs [33]. Owing to lifelong use and undesired effects of current allopathic drugs, research has been inclined towards the discovery of novel, curative, and safe therapeutic agents of natural origin for the management of CVDs [34]. Ethnopharmacology has been used as a successful tool to find new strategies and candidates for novel drugs and herbal medicines [35]. Since ancient times, medicinal plants have led to the development of highly effective tools for therapeutic purposes. Numerous studies have revealed that several plants have been pharmacologically evaluated based upon their empirical usage for the therapy of hypertension, and many of the currently marketed pharmaceutical drugs are synthesized based upon phytoconstituents isolated from these plants [1, 36]. Due to these reasons, in the current study, *Asphodelus tenuifolius* have been selected based upon the popular usage in diuresis and hypertension, for thorough pharmacological appraisal.

Data obtained in this study indicates a possible role of *A. tenuifolius* as an antihypertensive drug for the treatment of several CVDs. Our data showed the hypotensive and diuretic potential of *A. tenuifolius,* and according to our results, these effects appear to be attributed to the activation of muscarinic receptors. In fact, the hypotensive effect of aqueous fraction of *A. tenuifolius* (AS-AT) was not altered in the presence of L-NAME, indomethacin, or hexamethonium whereas atropine, a nonselective muscarinic acetylcholine receptor antagonist, significantly reduced the hypotensive response of AS-AT. In the vascular system, nitric oxide (NO) and prostacyclin (PGI2) regulate directly the arteriolar tone and consequently blood pressure levels. The main activator of NO and PGI2 synthesis in the vascular system is Ca2+. If Ca2+ levels rise, nitric oxide synthase (NOS) detaches from a protein called caveolin and is activated [37]. In addition, calcium catalyzes the activation of phospholipase A2, an important enzyme involved in the synthesis of PGI2. Currently, the role of acetylcholine on intracellular calcium levels is well established [38]. In vascular endothelium, M3 receptors may activate phospholipase C by increasing the inositol triphosphate (IP3) levels, which mobilizes Ca2+ from the cellular sarcoplasmic reticulum. Thus, substances capable of stimulating the M3 receptors may increase the release of NO and PGI2 and therefore reduce peripheral resistance and blood pressure. On the other hand, a recent study conducted by Tangsucharit et al. [39] has shown that the acetylcholine M1 and M3 receptors can also induce endothelium-independent vasodilation. In fact, removal of the endothelium in rat mesenteric arteries significantly decreased the levels of expression of M2 and M3, but not of M1. In mesenteric vascular beds denuded of the endothelium, ACh administration (10 and 100 nmol) caused long-lasting vasodilation, which was markedly blocked by treatment with highly selective antagonists, including pirenzepine (M1 antagonist) and 4-DAMP (M1 and M3 antagonists). These results suggest that the AChR muscarinic subtypes, mainly M1, are distributed by the rat mesenteric arteries and that the activation of M1 and/or M3, which may be located in the peptidergic nerves, releases calcitonin gene-related peptide (CGRP), causing vasodilation independent of the endothelium. Thus, as atropine is a nonselective muscarinic antagonist, it was able to block the effects of AS-AT, while L-NAME and indomethacin, which inhibit endothelial mediators (i.e., NO and PGI2), did not show this ability.

It has also been recognized that diuretics are the backbone of treatment for hypertension and edematous states characterized by surplus extracellular fluid [40]. Usually, diuretics enhance the excretion of large amounts of water and salts from the body in order to decrease blood volume and blood pressure and therefore reduce blood flow resistance. Despite the abundant availability of diuretics for use in humans, such medicines are related to different side effects majorly including various metabolic complaints, strengthening the significance of rummage around for new diuretic agents with more efficacy and fewer side effects. Therefore, in the current situation, new diuretic medicines are planned primarily from natural sources [7]. In fact, several medicinal plants display a number of pharmacological properties on the renal system, acting on different well-established targets such as nitric oxide-cGMP, renal carriers, carbonic anhydrase, prostaglandin-cAMP, and renin-angiotensin systems [41]. In the current study, crude extracts and fractions from *A. tenuifolius* induced significant diuretic and natriuretic effects in normotensive rats, with maximum diuretic response produced by AS-AT. Similar to hypotensive effects, AS-AT was subjected to further studies to evaluate the possible involvement of nitric oxide, acetylcholine, and prostaglandins in the renal action. In our study, we found that diuretic activity of AS-AT was reduced in the presence of muscarinic receptor blocker (atropine) whereas prostaglandin inhibitor (indomethacin) and NO synthesis inhibitor (L-NAME) did not alter the diuretic action. Thus, our findings suggest a muscarinic AChR-induced endothelium-independent vasodilation, which increases capillary blood flow leading to diuresis [42].

Oxidative stress has been the major underlying factor in the pathogenesis of various cardiovascular disorders like metabolic syndrome, diabetes, hypertension, kidney diseases, and heart failure owing to the rich supply of NADPH oxidase-induced ROS in vasculature and kidney. A high burden of ROS in the renal medulla will reduce the medullary blood flow and sodium excretion, resulting in high blood pressure and renal injury [43]. In the current study, various extract/fractions of *A. tenuifolius* seeds showed significant DPPH and nitric oxide (NO−) free radical scavenging ability, which is in line with the previously reported antioxidant potential of whole plant extract [9]. Therefore, we believe that the antioxidant effects presented by *A. tenuifolius* extract and their fractions may, directly or indirectly, contribute to the diuretic and hypotensive activities.

Preceding phytochemical studies of *A. tenuifolius* with GC-MS analysis have reported 20 different chemical constituents including some famous antioxidant compounds such as 5-hydroxymethylfurfural, 2, 3-dihydro-3, 5-dihydroxy-6-methyl-4H-pyran-4-one, cis-stilbene, and 2, 5-dimethyl-4-hydroxy-3(2H)-furanone (DMHF) [44, 45]. Moreover, many polyphenols, such as quercetin, rutin, apigenin, caffeic acid, and myricetin, were also identified in *A. tenuifolius* extract by HPLC-DAD. These polyphenols have been reported for their antihypertensive effects in various studies [46–50]. In this work, we analyzed the extract and fractions from *A. tenuifolius* by LC-DAD-MS, and seventeen compounds were described from AS-AT. The annotated compounds include organic acids, sugars, alkaloids, nucleoside, and glycosylated flavonoids. The molecular network revealed the components in the most active sample AS-AT compared to the other samples, and four major node cluster families were observed, which include glycosylated flavonoids and alkaloids. Thus, we believe that the presence of such constituents in *A. tenuifolius* extracts could be responsible for their hypotensive and diuretic potential. One of the limitations of our study was not being able to identify which compound found in AS-AT may be responsible for its cardiorenal activities. Despite this, we conjectured that this effect should not be attributed to only one representative but rather to coordinated and synergistic action of the various metabolites present in AS-AT.

## 5. Conclusion

This research supports and extends the traditional use of *A. tenuifolius* as a hypotensive and diuretic agent. The results showed that AS-AT from *A. tenuifolius* could present compounds responsible for hypotensive and diuretic activities with no signs of toxicity, and these effects could involve activation of muscarinic receptors.

## Figures and Tables

**Table 1:** Constituents identified from aqueous soluble fraction from *Asphodelus tenuifolius* by LC-DAD-MS/MS.

Peak	RT (min)	Compound	UV (nm)	MF	Positive mode (*m/z)*	
					MS [M + H]^+^	MS/MS
1	1.1	Hexitol	—	C_6_H_14_O_6_	183.0861	91
2	1.2	di-*O*-hexoside	—	C_12_H_22_O_11_	365.1037^a^	
3	1.3	Tri-*O*-hexoside	—	C_18_H_32_O_16_	527.1554^a^	365
4	1.3	Valine	—	C_5_H_11_NO_2_	118.0863	101
5	1.4	Unknown	—	C_16_H_26_N_2_O_11_	423.1618	254, 244, 224, 196
6	2.2	Adenosine	268	C_10_H_13_N_5_O_4_	268.1056	136, 119
7	2.8	Unknown	—	C_16_H_26_N_2_O_10_	407.1672	394, 369, 355, 309, 297, 271, 254
8	4.8	Peganine (vasicine)	285	C_11_H_12_N_2_O	189.1028	171, 143, 118, 140
9	5.0	di-*O*-hexosyl pegamine	280	C_23_H_32_N_2_O_11_	513.2060	351, 189, 118
10	7.0	di-*O*-hexosyl pegamine	280	C_23_H_32_N_2_O_11_	513.2067	351, 189, 118
11	7.9	Unknown	—	C_32_H_44_O_14_	675.2612	607, 417, 351
12	11.7	Tri-*O*-hexosyl quercetin	265, 355	C_33_H_40_O_22_	789.2087	465, 303
13	11.9	Tri-*O*-hexosyl luteolin	265, 342	C_33_H_40_O_21_	773.2158	449, 287
14	13.3	Unknown		C_16_H_23_NO_5_	310.1659	251, 207, 175, 147
15	14.7	6,8-Di-*C*-hexosyl apigenin	280, 332	C_27_H_30_O_15_	595.1637	505, 475, 433, 403, 379, 355, 337, 325
16	15.0	Unknown	—	C_26_H_35_NO_9_	506.2357	452, 413, 376, 298, 221
17	23.8	Tri-*O*-hexosyl *O*-deoxyhexosyl *O*-methyl apigenin	270, 338	C_40_H_52_O_24_	917.2947	447, 285, 270

RT: retention time; MF: molecular formula; ^a^: [M + Na]^+^.

**Table 2:** Total phenolic and flavonoid contents, total antioxidant capacity, and total reducing power of *A. tenuifolius*.

Sample	TPC (*μ*g GAE/mg DE	TFC (*µ*g QE/mg DE)	TAC (*μ*g AAE/mg DE)	TRP (*μ*g GAE/mg DE)	DPPH scavenging IC50 (*μ*g/ml)	Nitric oxide Scavenging IC50 (*μ*g/ml)
AT-Cr	256.64 ± 4.82	137.20 ± 2.37	193.05 ± 4.63	185.46 ± 3.77	174.6 ± 4.9	255.8 ± 6.9
AS-AT	190.51 ± 7.5	129.41 ± 8.2	176.85 ± 11.2	172.36 ± 9.0	274.8 ± 7.30	312.3 ± 8.97
BS-AT	187.2 ± 7.9	112.7 ± 6.3	165.9 ± 11.8	151.2 ± 8.7	398.4 ± 5.10	353.2 ± 11.4
DS-AT	92.1 ± 3.3	78.5 ± 6.2	83.7 ± 5.9	79.2 ± 8.3	453.7 ± 12.8	551.5 ± 13.8
AA	—	—	—	—	12.86 ± 2.10	225.1 ± 1.20

TPC: total phenolic contents; TFC: total flavonoid contents; TAC: total antioxidant capacity; TRP: total reducing power; GAE: gallic acid equivalent; QE: quercetin equivalent; AAE: ascorbic acid equivalent; DE: dry extract. Data values shown represent mean ± SEM (*n* = 3).

**Table 3:** Effect of *A. tenuifolius* extract/fractions on urine and electrolyte excretion.

Treatment (mg/kg)	Urine volume (ml/100 gm)			Na^+^ (mEq/L)	K^+^ (mEq/L)	pH
	2 h	4 h	6 h			
NS	1.42 ± 0.30	2.39 ± 0.05	3.5 ± 0.12	113.6 ± 13	40 ± 1.2	6.89 ± 0.12
HCTZ 10 mg/kg	2.8 ± 0.43	4.04 ± 0.34^*∗*^	7.14 ± 0.6^*∗∗∗*^	173 ± 11^*∗∗∗*^	56 ± 5.1^*∗*^	6.92 ± 0.72
AT-Cr 100 mg/kg	1.3 ± 0.25	2.54 ± 0.39	3.35 ± 0.5	128.3 ± 3	45.3 ± 4.8	6.65 ± 0.27
AT-Cr 200 mg/kg	2.44 ± 0.20	3.25 ± 0.42	5.5 ± 0.59^*∗∗∗*^	135 ± 3^*∗*^	52.3 ± 3.9	6.71 ± 0.18
AT-Cr 300 mg/kg	2.55 ± 0.38	3.44 ± 0.7	6.39 ± 0.8^*∗∗∗*^	160 ± 2 ^*∗∗∗*^	59.3 ± 8.6^*∗*^	6.69 ± 0.45
AS-AT 100 mg/kg	2.60 ± 0.44	4.03 ± 0.31^*∗*^	5.28 ± 0.4^*∗*^	121 ± 4	45 ± 2.3	6.71 ± 0.98
AS-AT 200 mg/kg	2.73 ± 0.46	4.57 ± 0.34^*∗∗*^	5.68 ± 0.52^*∗∗*^	132 ± 3^*∗*^	52.3 ± 6.1	6.78 ± 0.29
AS-AT 300 mg/kg	2.86 ± 0.10	4.96 ± 0.75^*∗∗*^	7.19 ± 0.7^*∗∗∗*^	221 ± 18^*∗∗∗*^	66 ± 11^*∗*^	6.91 ± 0.23
BS-AT 100 mg/kg	1.74 ± 0.06	2.31 ± 0.60	3.77 ± 0.26	117.6 ± 2.2	39.6 ± 7.09	6.70 ± 0.23
BS-AT 200 mg/kg	1.90 ± 0.10	3.05 ± 0.20	4.89 ± 0.22^*∗*^	125 ± 1.5	43 ± 2.5	6.69 ± 0.18
BS-AT 300 mg/kg	2.28 ± 0.17	3.21 ± 0.23	5.67 ± 0.22^*∗*^	131 ± 5^*∗*^	47.6 ± 2.9	6.74 ± 0.89
DS-AT 100 mg/kg	1.33 ± 0.17	2.81 ± 0.30	4.02 ± 0.47	121 ± 8	40.3 ± 7.5	6.56 ± 0.45
DS-AT 200 mg/kg	2.04 ± 0.69	3.12 ± 0.36	5.1 ± 0.49^*∗*^	130 ± 15^*∗*^	43.3 ± 4.9	6.61 ± 0.78
DS-AT 300 mg/kg	2.39 ± 0.88	3.44 ± 0.45	5.69 ± 0.69^*∗*^	135 ± 3^*∗*^	42.3 ± 3.9	6.68 ± 0.40

Results are stated as mean ± SEM where ^∗^ = *P* < 0.05, ^∗∗^ = *P* < 0.01, and ^∗∗∗^ = *P* < 0.001, as compared to normal saline- (NS-) treated group. HCTZ = hydrochlorothiazide, AT-Cr = crude extract of *A. tenuifolius*, AS-AT = aqueous soluble fraction of *A. tenuifolius*, BS-AT = butanol soluble fraction of *A. tenuifolius*, and DS-AT = dichloromethane soluble fraction of *A. tenuifolius*.

**Table 4:** Effect of *A. tenuifolius* extract/fractions on the diuretic index, Saluretic index, Lipschitz value, and excretion load.

Treatment (mg/kg)	Na/k	Diuretic index (DI)	Saluretic index Na^+^ (SI_Na_)	Saluretic index K^+^ (SI_K_)	Saluretic index (SI)	Lipschitz value (LV)	EL Na^+^	EL K^+^
NS	2.84	1.00	1.00	1.00	1	0.49	1.003	0.310
HCTZ 10 mg/kg	4.80	2.03	1.53	1.4	1.465	1.00	3.287	1.11
AT-Cr 100 mg/kg	2.83	0.954	1.129	1.132	1.130	0.46	1.19	0.42
AT-Cr 200 mg/kg	2.58	1.581	1.188	1.307	1.247	0.77	2.02	0.78
AT-Cr 300 mg/kg	2.69	1.820	1.411	1.482	1.445	0.86	2.72	1.008
AS-AT 100 mg/kg	2.68	1.503	1.065	1.125	1.095	0.78	1.69	0.63
AS-AT 200 mg/kg	2.93	1.617	1.161	1.307	1.484	0.70	1.98	0.784
AS-AT 300 mg/kg	3.348	2.04	1.948	1.65	1.75	0.78	4.20	1.254
BS-AT 100 mg/kg	2.95	1.077	1.035	0.99	1.012	0.528	1.23	0.414
BS-AT 200 mg/kg	2.90	1.39	1.10	1.075	1.087	0.68	1.69	0.58
BS-AT 300 mg/kg	2.75	1.62	1.15	1.19	1.17	0.79	2.06	0.749
DS-AT 100 mg/kg	2.57	1.144	1.070	1.182	1.125	0.56	1.33	0.52
DS-AT 200 mg/kg	2.74	1.566	1.237	1	1.118	0.77	2.10	0.60
DS-AT 300 mg/kg	2.59	1.621	1.188	1.307	1.247	0.79	2.02	0.784

NS = normal saline, HCTZ = hydrochlorothiazide, AT-Cr = crude extract of *A. tenuifolius,* AS-AT = aqueous soluble fraction of *A. tenuifolius*, BS-AT = butanol soluble fraction of *A. tenuifolius,* DS-AT = dichloromethane soluble fraction of *A. tenuifolius*. DI = urine volume of extract-treated group/urine volume of NS-treated group. SI_Na_ = urine Na^+^ excretion of extract-treated group/urine Na^+^ excretion of NS-treated group, SI_K_ = Urine K^+^ excretion of extract-treated group/urine K^+^ excretion of NS-treated group, SI = SI_Na_ + SI_K_ of treated group/SI_Na_ + SI_K_ of the control group, LV = urine volume of extract-treated group/urine volume of HCTZ-treated group, Excretion load (EL) = electrolytes concentration (mEq/l) × urinary flow (ml/min).

## Data Availability

All original data supporting the current study has been provided in this article.

## Abbreviations

ACE: Angiotensin-converting enzyme
AS-AT: Aqueous soluble fraction of *A. tenuifolius*
BS-AT: Butanol soluble fraction of *A. tenuifolius*
DS-AT: Dichloromethane soluble fraction of *A. tenuifolius*
AT-Cr: Crude extract of *A. tenuifolius*
CVD: Cardiovascular diseases
HCTZ: Hydrochlorothiazide
NO: Nitric oxide
ROS: Reactive oxygen species
TAC: Total antioxidant capacity
TFC: Total flavonoid contents
TPC: Total phenolic contents
TRP: Total reducing power
WHO: World Health Organization.
